# Prevalence and factors associated with PTSD among female urban slum dwellers in Ibadan, Nigeria: a cross-sectional study

**DOI:** 10.1186/s12889-021-11508-y

**Published:** 2021-08-12

**Authors:** Olutoyin Sekoni, Sumaya Mall, Nicola Christofides

**Affiliations:** 1 Department of Community Medicine, College of Medicine, University of Ibadan, Queen Elizabeth Road, Ibadan, Nigeria; 2 School of Public Health, University of the Witwatersrand, 27 St Andrews Road, Parktown, 2193 South Africa

**Keywords:** Post-traumatic stress disorder, Urban slum, Traumatic events, Nigeria

## Abstract

**Background:**

Little is known about the prevalence of and factors associated with PTSD among adult females in Nigeria, particularly those who live in slums. PTSD is a mental health condition that develops among some individuals who experience or witness a traumatic event. Several other factors could place individuals at heightened risk of PTSD including stress and comorbid mental disorders. Therefore, this study aimed to examine the prevalence and factors associated with PTSD among female urban slum dwellers in Ibadan, Nigeria.

**Methods:**

We conducted a cross sectional survey using multistage sampling of 550 women aged 18 and above from selected slums. Interviewer administered questionnaires were used to elicit information on experience of childhood trauma, recent stressors, intimate partner violence, other mental disorders, sociodemographic characteristics and PTSD. PTSD was measured using the Harvard Trauma Questionnaire (HTQ) which is based on DSM IV. A multivariable linear regression model was built to test associations between PTSD and independent variables.

**Results:**

The prevalence for PTSD was found to be 4.18% and the mean PTSD score was 5.80 ± 7.11. Sexual abuse in childhood, past year intimate partner violence and anxiety were significantly associated with higher PTSD scores. PTSD was not significantly associated with a history of recent stressors. Education, employment and marital status were not associated with PTSD however, age and wealth index showed marginal association with PTSD.

**Conclusion:**

The prevalence of PTSD among women living in Ibadan slums was relatively low. Both child sexual abuse and intimate partner violence can be prevented. We also recommend longitudinal studies to better understand risk and protective factors.

## Background

Post-Traumatic Stress Disorder (PTSD) is a disabling mental disorder consisting of three core symptoms: avoidance and numbing, re-experiencing and hyperarousal [1, 2]. PTSD can occur after the experience or witnessing of one or more Traumatic Events (TE) during childhood or adulthood [3, 4]. Traumatic events can include physical, sexual or emotional abuse during childhood [5, 6]. In adulthood these events include the experience or witnessing, of violent personal assault such as physical or sexual violence perpetrated by a partner, terrorism, serious illness or death of a loved one [7, 8]. The Diagnostic and Statistical Manual of mental disorders V (DSM V) highlights specific traumatic events at least one of which must be experienced prior to a diagnosis of PTSD [9].

Traumatic and stressful life events, can lead to the development of PTSD [10–13]. Traumatic stressors can include serious injury, illness or assault happening to a close relative or the death of a parent, child or spouse. The experience of several negative life events occurring during an individual’s lifespan is not unusual in the general population and this has been associated with negative mental health outcomes [12]. Even when the occurrence of negative life events are not strictly characterised as being of sufficient impact as to be traumatic, the degree to which an individual perceives such an event as traumatic can precipitate the development of mental health concerns such as PTSD [14].

PTSD has been found to be associated with mental disorders such as depression and anxiety [15]. This could be the result of co-morbidity or due to overlapping symptoms [16]. PTSD experienced together with other mental disorders, as well as PTSD on its own may contribute to a significant reduction in quality of life [17–19].

Research examining the prevalence and risk factors associated with PTSD suggests that it is associated with a range of socio-demographic characteristics [20–22]. Studies have found PTSD is more commonly reported among women [23–25]. Individuals with higher education levels are less likely to experience PTSD [26, 27]. However, there is inconsistent evidence with regards to marital status. Some studies suggest that marital status is protective against developing PTSD while others suggest that it increases the risk for PTSD [28–31]. Age has been found to play a role in the development of PTSD, with middle-aged women being at greatest risk [32, 33]. Unemployment after the experience of trauma has also been found to be significantly associated with PTSD [34].

Most studies examining PTSD have been conducted in the US and Europe (with earlier research focusing on service members and veterans) [35–37]. A few studies have been conducted on the African continent. In South Africa PTSD has been measured in a population-based mental health epidemiological survey, the South African Stress and Health Study (SASH). SASH suggested a lifetime and 12 month PTSD prevalence of 2.3 and 0.7% respectively while the risk of PTSD was found to be greatest among those who had lost a loved one [7]. Another population-based survey of adult women in one province in South Africa reported a prevalence of 11.6% [38]. High rates of PTSD (87%) were reported among female rape survivors surveyed from health facilities in South Africa [39]. The prevalence of PTSD among adolescents from low socioeconomic communities also in South Africa was about 6% [39, 40]. PTSD was reported among Eritrean refugees in Ethiopia and Rwanda among children of rape survivors [41, 42].

In Nigeria, where historically and currently there have been a wide range of TE and the prevalence of PTSD is thought to range widely between 2.7 and 66.7% [43–46]. Though reasons for such wide range variation in prevalence of PTSD are not known, it is possible that the use of varying study designs, tools and samples studied may have influenced this variation [47]. For example the cross-sectional study reported by Sheikh et al. reported a PTSD prevalence of 6.8%, while the case control study of Asuquo et al. reported a prevalence of 41.3% [43, 45]. Predictors of PTSD in Nigeria include a wide spectrum of events e.g. political and ethno-religious violence to road traffic accidents [43–46, 48]. However, slum environments tend to be associated with a greater number of TE, therefore the risk of developing PTSD in such settings may be higher [49–51]. To our knowledge, there are limited prevalence data for PTSD from community-based samples and poor urban slum settings in Nigeria.

Slum areas in Ibadan, which is the setting of this study, are located in the central and oldest parts of the city and represent areas of little or no identifiable sanitation facilities or social infrastructure, physical deterioration, and very high population density [52, 53]. The slums developed in the wake of the colonial era which was followed by an increase in economic activities making it possible for individuals to attain economic independence at earlier ages and as a result the desire to build personal property [54]. In a bid to remain within the extended family setting, such houses could only be built within the open space of the family compound. This ultimately has led to wide spread overcrowding and poor access to infrastructure such as water, electricity and sewerage due to the un-planned nature of the neighbourhoods and almost non-existent road networks [55, 56].

The purpose of this study was to estimate the prevalence of PTSD, and to describe associated factors including socio demographic characteristics, experience of childhood trauma, intimate partner violence, recent stressors, as well as comorbid mental disorders among women in a slum setting in South West Nigeria. Our study addresses gaps in the literature on PTSD with particular reference to trauma across the life course and mental health in community samples as well as among women in deprived circumstances such as a slum setting in Ibadan, Nigeria.

The results potentially contribute to the body of literature on PTSD among women in low income urban neighbourhoods and the role that neighbourhood context plays in the development of PTSD which are research gaps that have been highlighted by other researchers [57]. It will also contribute to our understanding of the prevalence of comorbid mental disorders in such contexts [58, 59].

## Methods

### Participants

A community based cross-sectional household survey was conducted in slum areas of Ibadan in Oyo State. Participants were adult women above the age of 18 years who were resident members of the selected households during the period October to November 2018. Regular residence was defined as spending at least four nights a week in the household for at least 1 year [60].

### Procedure

Six female research assistants with tertiary education were recruited and trained. Training of research assistants took place over 5 days and covered data collection and ethical considerations relevant for the study. These included method of administration of questionnaires, review of questions for completeness and maintenance of ethical standards with particular emphasis on the sensitive nature of the questions. The training also included the World Health Organization requirements for collecting data for trauma research [61]. Training on the use of the Open Data Kit (ODK) was also carried out to teach research assistants how to use the software and upload the data once obtained. ODK is a mobile technology that enhances the data collection and data management process by ensuring real time data entry through the use of the internet. It was developed at the University of Washington and has been found to be an effective data collection tool [62, 63]. Role plays were utilised in order enhance skills and evaluate the training.

A sample of 550 eligible women were selected based on a multistage sampling technique. Simple random sampling was used to select five of thirteen slum settlements in Ibadan through the use of a table of random numbers. The selected slums were: Beere, Oje, Oritamerin/Agbeni, Ojaba and Mapo located within the central core of the city occupied by the indigenous people of Ibadan and are also known as “traditional slums ”[52]. Enumeration Areas (EAs) as demarcated by the National Population Commission [64] were identified by using already existing maps prepared by the Commission. One third of the EAs were selected from each of the five slums using a table of random numbers. This yielded a total of 42 EAs. However this number was later increased to 68. This ensured that there were sufficient households available per EA to select one eligible respondent per household while adhering to the required sample size. This approach has been utilised in the conduct of other population based surveys on violence research in order to ensure safety of both the respondent and the interviewer [61]. The Nigerian Demographic and Health Survey (NDHS) indicates 48 households per EA [60]. However this figure was not specified for a slum area where higher number of households would be expected. We assumed an average of 60 households per EA (four households per dwelling in 15 dwellings per EA). One household was selected randomly by balloting per house where there were at least two households. One person in the selected household was selected randomly by balloting. Data collection was carried out through the use of face to face interviews by the research assistants using standardized questionnaires uploaded on mobile devices with ODK software.

### Measures

#### Outcome variable

The outcome variable for this study was PTSD assessed using the Harvard Trauma Questionnaire (HTQ) which is based on DSM-IV PTSD criteria [65]. The HTQ is a reliable screening instrument that has been widely used and is acceptable across cultures [65–67]. The trauma symptom items of the HTQ were used in this study. Items included an individual’s perception of their ability for daily functioning. For example, “*feeling detached or withdrawn from people*” and”, “*feeling as though the event is happening again”.*

The HTQ utilizes a 4 point Likert scale of 16 items with responses of “not at all”, “a little bit”, “quite a bit” or “extremely” to determine the occurrence of post-traumatic symptoms within the previous week [65, 68]. A score of 1 was assigned to the responses of “not at all”, a score of 2 was assigned to the responses of “a little bit”, a score of 3 was assigned to the responses of “quite a bit” and 4 was assigned to responses of “extremely” The minimum score was 1 and the maximum possible score was 64. The sum of the items were then divided by the total number of items. A cut point of 2.0 was used in interpreting the score which has been recommended for use when doing community based research as opposed to the cut point of 2.5 recommended for use among clinic based population samples [65, 67, 69]. We adopted this lower cut point as population characteristics have been suggested to influence the performance of the HTQ with differences seen among highly traumatised populations as opposed to those that are not as highly truamatised [67]. Based on this, the outcome variable was dichotomized as either ‘No PTSD symptoms’ or as having ‘PTSD symptoms’. We found good internal consistency with a Cronbach’s alpha of 0.88.

#### Explanatory variables

Experiences of childhood trauma were assessed using the Childhood Trauma Questionnaire (CTQ). The CTQ is an instrument consisting of 28 items covering a range of childhood experiences [70]. Domains include emotional abuse and neglect, physical abuse and neglect and sexual abuse [70, 71]. For the purposes of the analyses presented in this paper we used only the sexual, physical and emotional abuse sub-scales as they are more likely to be associated with PTSD [72, 73]. The CTQ has been used previously on the African continent [74, 75]. We found good internal consistency with a Cronbach’s alpha of 0.82. Examples of items on the CTQ include: “*People in my family called me things like ‘stupid* ’*, ‘lazy* ’ *or ‘ugly* ’, “*I got hit so hard by someone in my family that I had to see a doctor or go to the hospital*” , “*Someone tried to touch me in a sexual way or tried to make me touch them*” . Each subscale was assessed as a dichotomous variable with any affirmative answer to an item as an experience.

Intimate partner violence was assessed using the WHO Multi-country Study on Women’s Health and Domestic Violence Core Questionnaire [61]. Experiences of intimate partner violence were asked for within the various domains of physical and sexual abuse. The internal consistency of the scale was good at a Cronbach’s alpha of 0.84. In our analysis, we identified past year experience of intimate partner violence with variable responses being having had an experience of intimate partner violence in the year prior to the study or not having had an experience of intimate partner violence in the year prior to the study.

Recent stressors were assessed using the Life Events Questionnaire [76]. The scale considers recent life events that tend to be stressful and threatening and have occurred within the preceding 6 months. It is a 12 item self- report questionnaire which assesses the quality of life of an individual before, during and after the occurrence of a stressful life event. We utilised four items of the twelve item questionnaire as these four items were the only ones that fit the PTSD criteria for a traumatic experience [77, 78]. The items are as follows: “*Did you suffer serious illness, injury or assault?*” *,* “*Did your parent, child or spouse die?*” *,* “*Did a serious illness, injury or assault happen to a close relative?*” and “*Did a close family friend or another relative (aunt, cousin, grandparent) die?*” For each item a “yes” (with a score of 1) or “no” (with a score of 0) response was given which has been identified as a valid method of assessment [79]. The frequency of each of the items has been presented. In addition to this, a dichotomous score was computed from the total sum of all the items and was categorized into “no stressors” or “any stressors”.

Depression and anxiety was assessed using the DASS-21 (Depression, Anxiety and Stress Scale-21) which has been used previously in Africa [80]. It contains three subscales: one subscale for depression, one for anxiety and one for stress. Each subscale is composed of 7 separate items using a 4 point Likert scale with the respondent identifying the extent to which each of the statements on the instrument applied over the past 1 week [81]. Examples of items on the depression scale include; “*I found it difficult to work up the initiative to do things*” and “*I felt that I had nothing to look forward to*”.

Examples of items on the anxiety scale include;” I experienced trembling (e.g in the the hands)” and “I was worried about situations in which I might panic and make a fool of myself”.

Scores for each scale are calculated by summing the scores for the items on each scale. The cutoff values serve as a means of interpretion with respect to the dimensions of severity. Responses for each of the scales are indicated as 0 (did not apply to me at all), 1(applied to me to some degree, or some of the time), 2 (applied to me to a considerable degree or a good part of time), 3 (applied to me very much or most of the time). Scores on the DASS 21 are multiplied by 2 to give the final score which is categorized accordingly into “normal”, “mild”, “moderate”, “severe”, and “extremely severe”.

Due to very small numbers of participants with DASS scores in the moderate and severe categories and none in the extremely severe category, we collapsed categories for the depression and anxiety scales. We generated a dichotomous variable for depression and anxiety with categories of “no anxiety” representing the intial “normal” and “mild” and “anxiety” represented by collapsing the initial categories of “moderate”, “severe” and “extremely severe”. No participants had stress scores that were in the “moderate”, “severe” or “extremely severe” category so we did not include the assessment of stress in this study.

Validity, reliability and internal consistency of the DASS has been found to be satisfactory across various populations of both clinical and non - clinical samples [82–84]. We found satisfactory internal consistency with a Cronbach’s alpha of 0.73.

Sociodemographic characteristics: included age, educational status, marital status, employment status, occupation and wealth index. Age was categorized into three age groups as follows: 18–29 years, 30–44 years and age 45 years or older. Educational status was categorized as none- for those who had not undergone any form of formal education, primary- which represents the first 6 years of schooling and secondary- which represents the subsequent 6 years of schooling. In our study, marital status was categorized as “married”- for those in a formal marriage union, “single”- for those not in any formal marriage union and “others”- for those that did not fall into the category of married or single such as those who were widowed, separated or divorced. Employment status was determined by whether or not the respondent had been employed within the last 3 months or not with responses being “yes” or “no”. Respondents were categorized into three major occupations: trader, artisans and professionals. Our assessment of wealth was done utilizing the 11 item Simplified Asset Index, a previously published tool which assesses the relative wealth profile of urban dwellers in comparison to other urban dwellers making it a more accurate assessment of wealth for the urban poor [85]. The Simplified Asset Index is based on Demographic and Health Survey (DHS) data. The DHS has been implemented in more than 90 countries since 1984 [86] and allows for reliable sub-group analyses in urban areas. The index is a shorter country specific alternative to the original DHS wealth index variables but has been found to equally effective and easier to administer [85]. Examples of items from the Simplified Asset Index include; “*What type of fuel does your household mainly use for cooking*?” and “*Does any member of this household have a bank account?”*

The original Simplified Asset Index is divided into 5 quintiles but due to small cell numbers we had to merge these into three. The three urban wealth index categories were assigned as follows: the lowest category 1 being “poorest”, the middle category 2 being “poor” and the highest category 3 being “slightly wealthier”.

The instrument was translated to the local language [Yoruba] and back translated to ensure that original meanings were preserved and was pre-tested in an enumeration area within a slum area that had not been selected for the study. Such a process of translation has been described by other researchers [87]. Based on the feedback received, we made corrections to the questionnaire.

Ethical approval for this study was sought from the University Of Witwatersrand Faculty Of Health Science Human Ethics Committee as well as the Ethics Review Board of the Oyo State Ministry of Health, Oyo State, Nigeria.

#### Data analysis

Data were analyzed using STATA Version 14.0 software (Stata Corp., College Station, TX, USA). There were no missing data on the outcome measure of interest and hence no case dropping was implemented. We adjusted for the cluster sampling during analysis using the *svyset* command in STATA. We also weighted the data to ensure that it was representative of the Ibadan slums using the command *pweight*. The outcome variable under consideration was the mean PTSD score while the explanatory variables included the experience of childhood trauma (sexual, physical and / or emotional abuse), sexual and /or physical intimate partner violence, recent stressors, depression, anxiety and sociodemographic characteristics. T-tests were used to compare mean PTSD symptomatology scores with explanatory variables such as child sexual abuse, intimate partner violence, depression and anxiety which were dichotomous variables. Oneway ANOVA was used for categorical variables such as educational attainment, marital status and wealth index. Subsequently, a multivariable linear regression analysis was built to examine associations between PTSD score and explanatory variables which were associated in the univariate analyses or considered theoretically important based on the literature. Statistical significance was set at *p* < 0.05.

## Results

All 550 women in selected households agreed to participate in the study giving a 100% response rate. This high response rate is not uncommon in the Ibadan context. The median age of respondents was 40 years with an interquartile range of 30–55 years. About a fifth had never attended school (22.4%). Almost two thirds were married or cohabiting with a partner (67.8%). The majority of the sample had been employed 3 months prior to the interview (77.3%) and came from a household that was in the poor and poorest urban wealth categories (71.6%). Twenty three participants from our sampled population reported PTSD symptoms and this yielded a PTSD prevalence of 4.18%.

With respect to the univariate association with PTSD, being a woman from a household in the poorest category was associated with the occurrence of PTSD even though the association was not strong. No other sociodemographic characteristics were associated with PTSD (Table 1).

**Table 1:** Associations between sociodemographic characteristics and PTSD

	PTSD symptoms n (%)	No PTSD symptoms n (%)	*P* value
**Age at last birthday**			
18–29	5 (21.74)	132 (25.05)	
30–44	10 (43.48)	178 (33.78)	0.63
≥ 45	8 (34.78)	217 (41.18)	
**Educational status**			
None	6 (26.09)	117 (22.20)	
Any Primary	7 (30.43)	158 (29.98)	0.74
Some Secondary	1 (4.35)	66 (12.52)	
Completed secondary/Tertiary	9 (39.13)	186 (35.29)	
**Marital status**			
Married/Cohabiting	15 (65.22)	358 (67.93)	
Single/Never married	3 (13.04)	40 (7.59)	0.59
Separated/widowed/divorced	5 (21.74)	129 (24.48)	
**Employed in the last 3 months**			
No	8 (34.78)	117 (22.20)	
Yes	15 (65.22)	410 (77.80)	0.15
**Wealth index**			
Poorest	13 (56.52)	171 (32.45)	
Poor	5 (21.74)	205 (38.90)	0.05^a^
Slightly wealthier	5 (21.74)	151 (28.65)	

^a^Marginal finding

The prevalence of the trauma experiences during childhood ranged from 8.9% (sexual abuse), 50.4% physical abuse and 70.4% emotional abuse. Women without a history of child sexual abuse were less likely to report PTSD symptoms and this was statistically significant. The experience of recent stressors was not associated with reports of PTSD symptoms among the women. Women who were not depressed and not anxious were also significantly less likely to report PTSD symptoms (Table 2).

**Table 2:** Association between traumatic events, mental disorders and PTSD

Childhood trauma	PTSD symptoms n (%)	No PTSD symptoms n (%)	*P* value
**Sexual abuse**			
None	15 (65.22)	486 (92.22)	0.000*
Any sexual abuse	8 (34.78)	41 (7.78)	
**Emotional abuse**			
None	4 (17.39)	159 (30.17)	0.246^a^
Any emotional abuse	19 (82.61)	368 (69.83)	
**Physical abuse**			
None	8 (34.78)	265 (50.28)	0.146
Any physical abuse	15 (65.22)	262 (49.72)	
**Recent Stressors (Items)**			
**Suffered serious illness, injury or assault**			
No	18 (78.26)	450 (85.39)	
Yes	5 (21.74)	77 (14.61)	0.34
**Serious illness, injury or assault happened to a close relative**			
**No**	21 (91.30)	492 (93.36)	
Yes	2 (8.70)	35 (6.64)	0.66^a^
**Parent, child or spouse died**			
**No**	20 (86.96)	469 (88.99)	
**Yes**	3 (13.04)	58 (11.01)	0.73^a^
**A close family friend or another relative (aunt, cousin, grandparent) died**			
**No**	22 (95.65)	454 (86.15)	0.19^a^
**Yes**	1 (4.35)	73 (13.85)	
**Recent stressors (Total)**			
No stressors	14 (60.87)	375 (71.16)	
Any stressors	9 (39.13)	152 (28.84)	0.28
**Past year Intimate Partner Violence**			
No	18 (78.26)	441 (83.68)	
Yes	5 (21.74)	86 (16.32)	0.49
**Depression**			
No depression	22 (95.65)	524 (99.43)	0.15^a^
Depression	1 (4.35)	3 (0.57)	
**Anxiety**			
No anxiety	16 (69.57)	496 (94.12)	0.00*
Anxiety	7 (30.43)	31 (5.88)	

^a^Fishers exact *p < 0.05

We conducted a regression analysis to examine the relationship between PTSD symptoms, traumatic events and mental disorders while controlling for sociodemographic characteristics. Child sexual abuse, recent intimate partner violence and symptoms of anxiety were all associated with a higher PTSD score. For age and wealth status, there were marginal associations with a higher PTSD score. The age of women who were 30–44 compared to those who were in the 18–29 year age group were marginally more likely to have higher PSTD scores. Women in the slightly wealthier category were less likely to report PTSD symptoms compared to the poorest women. PTSD was not significantly associated with a history of other recent stressors among the sample (Table 3).

**Table 3:** Multivariable linear regression model of PTSD symptoms and traumatic events and mental disorders adjusting for socio demographic characteristics (*N* = 550)

Variable	Beta	95% CI	*P* value
**Childhood trauma**			
**Sexual abuse**			
None	Ref		
Any sexual abuse	3.82	1.75–5.88	0.00
**Emotional abuse**			
None	Ref		
Any emotional abuse	0.81	−0.56- 2.19	0.24
**Physical abuse**			
None	Ref		
Any physical abuse	0.55	−0.70-1.82	0.38
**Recent Stressors (Total)**			
No stressors	Ref		
Any stressors	1.08	−0.17-2.34	0.09
**Past year Intimate Partner Violence**			
No	Ref		
Yes	1.83	0.20–3.46	0.02
**Depression**			
No Depression	Ref		
Depression	2.08	−4.71-8.88	0.54
**Anxiety**			
No Anxiety	Ref		
Anxiety	6.38	4.09–8.67	0.00
**Age at last birthday**			
18–29	Ref		
30–44	1.68	−0.04- 3.41	0.05^a^
≥ 45	0.95	−0.99-2.90	0.33
**Educational status**			
None	Ref		
Any Primary	−0.05	−1.94-1.83	0.95
Some Secondary	−0.41	−2.77-1.94	0.73
Completed secondary/Tertiary	0.24	−1.78-2.27	0.81
**Marital status**			
Married/Cohabiting	Ref		
Single/Never married	1.65	−0.92-4.22	0.20
Separated/widowed/divorced	0.87	−0.88-2.63	0.32
**Employed in the last 3 months**			
No	Ref		
Yes	0.46	−1.96-1.03	0.54
**Wealth index**			
Poorest	Ref		
Poor	−0.73	−2.17-0.70	0.31
Slightly wealthier	−1.53	−3.15-0.07	0.06^a^

^a^Marginal findings CI Confidence Interval

## Discussion

Our study set out to estimate the prevalence of PTSD and associated factors among women in a slum setting in South West Nigeria. We found a prevalence of 4.18% for PTSD with only 23 women among our sample of 550 women reporting symptoms of PTSD. Sexual abuse during childhood, recent experience of intimate partner violence as well as anxiety were significantly associated with PTSD. A history of recent stressful life events was not associated with PTSD. None of the sociodemographic characteristics considered (age, education, marital status or wealth index) were associated with PTSD. However, in the multivariable linear regression analysis there were marginal associations with age and wealth index only.

We observed low prevalence of PTSD among our respondents despite their precarious circumstances in the slum environment, which included high levels of adverse life events, very limited resources and services. In urban slums elsewhere, researchers have described greater prevalence of PTSD among residents of as a result of the adversity experienced within such settings. Mbwayo et al. reported a PSTD prevalence of 15.4% among respondents in urban slums in Kenya [88] while Ndugu et al. reported 21% among women in informal settings in South Africa [89]. A high prevalence of 29 and 20% was also reported among women in low income neighbourhoods in the United States [51, 59]. There is evidence in literature to suggest that resilience may play a role in moderating the effect of trauma and subsequent PTSD experienced. This has been seen among populations that are at particular risk for PTSD such as police officers and fire-fighters [90, 91], as well as among undergraduate women [92]. Resilience may explain the low prevalence we observed.

We found that a history of sexual abuse in childhood was associated with PTSD in adults, a finding which has been reported previously [93–95]. Child sexual abuse has also been reported widely on the African continent [96–100]. We did not find an association between physical and emotional abuse during childhood and PTSD although other studies have reported an association. McLaughlin and colleagues who analysed data from the World Mental Health Surveys suggest that 4 childhood adversities out of a potential of 12 were associated with the onset of PTSD. Childhood physical abuse and sexual abuse were amongst these [101]. In another analysis from the WMHS led by Koenen and colleagues, the odds of lifetime PTSD among trauma exposed adults was 2.5 [102].

The experience of sexual and / or physical intimate partner violence represents a trauma in adulthood. We found that recent intimate partner violence was significantly associated with a higher PTSD symptoms among our sample. Research from other sub-Saharan African countries has reported an association [38]. Mahenge and colleagues in Tanzania also found higher levels of PTSD among pregnant women who had experienced intimate partner violence [103]. A multicountry study among university students from 25 countries (7 countries from sub-Saharan Africa) also suggested that PTSD was associated with intimate partner violence in all of the sub-Saharan African countries [104].

We found an association between PTSD and anxiety. This association has been reported by other authors [105, 106]. There are two possible explanations for this finding: it is possible that women with anxiety may be more susceptible to developing PTSD (or vice versa) or it could be that they share common symptoms [16]. There may be an overlap of symptoms of PTSD and the spectrum of both anxiety and depressive disorders which may mask anxiety and mood symptoms and as such must be borne in mind when assessing for PTSD [16]. A bidirectional association between anxiety and PTSD has been suggested in the literature with some researchers reporting that anxiety predisposes to PTSD in the face of trauma while others indicate that PTSD is a risk factor for anxiety [107, 108]. It is clear that anxiety occurs very commonly with PTSD and as such treatment measures for PTSD should factor in the need to screen for as well as treat this co-morbidity.

We did not find any association between the experience of stressful life events such as serious illness, injury or assault or death of a relative within the preceding 6 months and symptoms of PTSD. However, other studies conducted in different settings have shown that stressful life events can contribute to the development of PTSD [13, 109, 110]. The findings with respect to the association between sociodemographic characteristics and PTSD have been mixed in the literature. Age has generally not been associated with PTSD as has been reported by authors in Africa [7, 48, 111, 112]. However, Kobayashi in the United States reported an association between the middle aged subgroup of women and PTSD, with middle aged women being more likely to have PTSD compared to younger and older women after controlling for potential confounders [33]. Our results are similar to those of Kobayashi, with women aged 30 to 44 having statistically significant higher PTSD scores than women aged 18- to 29. Middle aged women are thought to carry the greater burden and stress of care- giving for both the younger and older individuals within the community and a disruption of that role due to trauma can be distressing to them and make them more likely to suffer from anxiety and depression [113]. Some authors have reported that educational level is associated with PTSD with individuals with higher levels of education less likely to develop PTSD [20, 23, 114–116]. We did not find an association between education and PTSD in our sample. Other researchers in Africa have reported that education level, employment status, religious affiliation and marital status were not associated with PTSD [117].

The aetiology of PTSD is multifactorial and differs depending on context [118]. The context of our study may have protected participants from exposure to trauma and the development of PTSD. The particular slum environment in urban Ibadan where the study took place is stable and homogenous. Most participants belonged to one ethnic group and had lived in the area for most or all of their lives. Literature has suggested that living in neighborhoods that have greater ethnic homogeneity is protective against mental disorders [119, 120]. This has been described as a function of the perception of being in similar socioeconomic circumstances to everyone else in the environment and with it the a higher acceptance of income inequality and less mental stress [120]. With a majority of our slum sample falling within the same lower wealth categories, this explanation may not be out of place.

It is evident in that not everyone exposed to trauma develops PTSD. The results of our study among this sample has highlighted this clearly with unexpectedly low levels of PTSD in spite of a substantial history of childhood trauma as well as less than optimal living conditions of a slum setting. This suggests the need for further research to explore resilience to PTSD and individual and community-based factors that may provide protection from developing symptoms of PTSD in the slum setting in Ibadan, Nigeria. Factors such as social connectedness, self – esteem and social support have been found to be protective against the development and severity of PTSD [121–124].

The results of the study need to be interpreted in the light of several limitations. From a study design standpoint, the cross-sectional nature of our study precludes conclusions about the temporal relationship between intimate partner violence, anxiety and PTSD. The HTQ was developed in the United States and it is possible that cultural differences between our study population and the original population for which it was designed could have resulted in underreporting of PTSD symptoms. Several studies have suggested that there might be cultural explanations for mental disorders such as depression and that language and culture shape views of illness [125, 126].

We also cannot be sure whether translation of instruments might have resulted in the loss of meaning of items measuring mental disorders as has been suggested by seminal work on translation and mental health care in South Africa, a culturally and linguistically diverse society [127, 128].

Reports of childhood trauma and stressors could be affected by recall bias with participants underreporting the occurrence. However, retrospective accounts of trauma have been found to be acceptable for PTSD research [129]. Certain kinds of traumatic events were not measured in this study. We did not ask about experiences of witnessing a murder or witnessing of rape, to name a few. Our study was not sufficiently powered because of our expectation of a higher prevalence of PTSD in this sample based on previous research that had indicated higher prevalence of PTSD in a slum setting.

## Conclusions

Our findings shed light on the low levels of PTSD symptoms among this community based sample of poor urban women. However, there was a significant relationship between the experience of childhood trauma and PTSD. In addition, intimate partner violence and anxiety were also associated with PTSD. Our findings add to the literature which suggests that the consequences of early childhood experiences of trauma especially childhood sexual abuse and intimate partner violence can have quite serious mental health consequences including PTSD. These results speak to the urgency to prevent both childhood abuse as well as intimate partner violence. Policy and actions geared towards these two issues would be particularly important as both are preventable and ultimately could reduce the incidence of PTSD.

## Data Availability

The data that support the findings of this study are available from the corresponding author upon reasonable request.

## Abbreviations

CI: Confidence Interval
EAs: Enumeration Areas
DASS: Depression Anxiety and Stress Scale
PTSD: Post-Traumatic Stress Disorder
TE: Traumatic Events
ODK: Open Data Kit
