# Correction to: Prevalence and factors associated with PTSD among female urban slum dwellers in Ibadan, Nigeria: a cross-sectional study

**DOI:** 10.1186/s12889-021-11722-8

**Published:** 2021-09-27

**Authors:** Olutoyin Sekoni, Sumaya Mall, Nicola Christofides

**Affiliations:** 1 Department of Community Medicine, College of Medicine, University of Ibadan, Queen Elizabeth Road, Ibadan, Nigeria; 2 School of Public Health, University of the Witwatersrand, 27 St Andrews Road, Parktown, 2193 South Africa

**Correction to: BMC Public Health 21, 1546 (2021)**

**http://orcid.org/10.1186/s12889-021-11508-y**

It was highlighted that in the original article [1] the Funding section was incorrect. This Correction article shows the correct Funding section. The original article has been updated.

**Funding**

OS was supported by the Consortium for Advanced Research Training in Africa (CARTA). CARTA is jointly led by the African Population and Health Research Center and the University of the Witwatersrand and funded by the Carnegie Corporation of New York (Grant No—G-19-57145), Sida (Grant No:54100113), Uppsala Monitoring Centre and the DELTAS Africa Initiative (Grant No: 107768/Z/15/Z). The DELTAS Africa Initiative is an independent funding scheme of the African Academy of Sciences (AAS)‘s Alliance for Accelerating Excellence in Science in Africa (AESA) and supported by the New Partnership for Africa’s Development Planning and Coordinating Agency (NEPAD Agency) with funding from the Wellcome Trust (UK) and the UK government. The statements made and views expressed are solely the responsibility of the Fellow.

SM has been supported by a Claude Leon award for early career researchers and a self-initiated research fellowship from the South African Medical Research Council (2018–2020).

The funders had no role in the design of the study, collection, analysis, and interpretation of data nor in writing the manuscript.
